# Relation between Ga Vacancies, Photoluminescence, and Growth Conditions of MOVPE-Prepared GaN Layers

**DOI:** 10.3390/ma15196916

**Published:** 2022-10-05

**Authors:** Alice Hospodková, Jakub Čížek, František Hájek, Tomáš Hubáček, Jiří Pangrác, Filip Dominec, Karla Kuldová, Jan Batysta, Maciej O. Liedke, Eric Hirschmann, Maik Butterling, Andreas Wagner

**Affiliations:** 1 Institute of Physics CAS, Cukrovarnická 10, 162 00 Prague, Czech Republic; 2 Faculty of Mathematics and Physics, Charles University, V Holešovičkách 2, 180 00 Prague, Czech Republic; 3 Faculty of Nuclear Sciences and Physical Engineering, Czech Technical University, Břehová 7, 115 19 Prague, Czech Republic; 4 Institute of Radiation Physics, Helmholtz-Zentrum Dresden-Rossendorf, Bautzner Landstr. 400, 01328 Dresden, Germany

**Keywords:** GaN, defects, positron annihilation spectroscopy, photoluminescence, MOVPE

## Abstract

A set of GaN layers prepared by metalorganic vapor phase epitaxy under different technological conditions (growth temperature carrier gas type and Ga precursor) were investigated using variable energy positron annihilation spectroscopy (VEPAS) to find a link between technological conditions, GaN layer properties, and the concentration of gallium vacancies (V_Ga_). Different correlations between technological parameters and V_Ga_ concentration were observed for layers grown from triethyl gallium (TEGa) and trimethyl gallium (TMGa) precursors. In case of TEGa, the formation of V_Ga_ was significantly influenced by the type of reactor atmosphere (N_2_ or H_2_), while no similar behaviour was observed for growth from TMGa. V_Ga_ formation was suppressed with increasing temperature for growth from TEGa. On the contrary, enhancement of V_Ga_ concentration was observed for growth from TMGa, with cluster formation for the highest temperature of 1100 °C. From the correlation of photoluminescence results with V_Ga_ concentration determined by VEPAS, it can be concluded that yellow band luminescence in GaN is likely not connected with V_Ga_; additionally, increased V_Ga_ concentration enhances excitonic luminescence. The probable explanation is that V_Ga_ prevent the formation of some other highly efficient nonradiative defects. Possible types of such defects are suggested.

## 1. Introduction

Optimization of methods for high-quality GaN growth in the 1990s paved the way for GaN-based power-efficient optoelectronic devices [1]. Highly efficient InGaN blue and white LEDs are now commercially available [1]. Thanks to the high polarization coefficient and ability to form heterostructures, AlGaN/GaN high electron mobility transistors can be manufactured, and these can be found in many applications these days [2]. Other applications of GaN-based devices include UV detectors [3], photovoltaic cells [4], and scintillators [5]. Despite extensive research on both the application and basic properties of GaN and related materials in the past three decades, several fundamental questions remain unanswered. One of them is the role or even the presence of Ga vacancies in GaN.

Defects related to Ga vacancies (V_Ga_) in GaN are extremely important for optoelectronic applications since they are believed to be the main cause for the nonradiative recombination of carriers [6]. Other scholars have proposed that V_Ga_ are responsible for yellow band luminescence and form nonradiative centres if they are in a complex with donors [7,8,9]. V_Ga_-related defects deteriorate the frequency properties of high electron mobility transistors (HEMTs), since trapping and releasing a charge in deep levels is a rather slow process, decreasing the cut-off frequency. V_Ga_ can also decrease electron channel mobility. Another problem of these defects is shortening of the device lifetime, since vacancies enhance the diffusion of atoms in the structure, which can lead to the decomposition of quantum wells (QWs) [10] or the back-diffusion of Mg atoms from the GaN capping layer through the AlGaN barrier [11,12] to the channel region in HEMT structures. Although V_Ga_ and their complexes are very important defects in nitrides, there is a lack of knowledge about the link between formation of these defects and the technological parameters of the preparation of high-quality epitaxial layers by metalorganic vapor phase epitaxy (MOVPE), as well as the influence of vacancies on semiconductor properties.

## 2. Materials and Methods

The studied samples were prepared by Aixtron CCS 3 × 2 MOVPE apparatus with dynamic adjustment of showerhead height. The following precursors were used for epitaxy: trimethylgallium (TMGa), triethylgallium (TEGa), and ammonia (NH_3_). The set of prepared samples is specified in Table 1.

**Figure 1:**
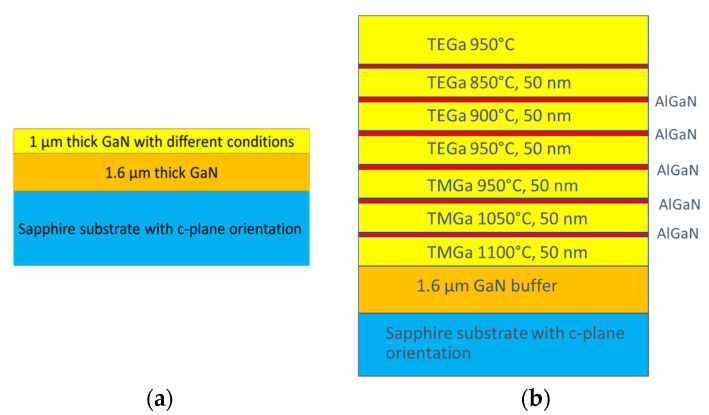
(**a**) Structure of samples prepared for VEPAS and PL measurement, (**b**) Structure of samples prepared for SIMS.

Samples were prepared with the aim of finding a link between vacancy formation and some MOVPE technological parameters, namely temperature, type of carrier gas, and type of precursor. Growth of samples was always repeated in both atmospheres, H_2_ or N_2_. The growth temperature was changed from 850 °C to 1100 °C. TEGa was used for the growth in the temperature range of 850–950 °C; growth at higher temperatures with this precursor was not possible due to strong adduct formation. TMGa was used at the higher temperature range of 950–1100 °C. Growth at lower temperatures from TMGa was avoided due to strong carbon incorporation. Four samples, TEN3, TEH3, TMN1, and TMH1 were prepared at the same temperature (950 °C) to compare only the influence of precursor type. All samples had a 1 µm thick GaN layer prepared with relevant technological parameters. Their simple structure is shown in Figure 1a.

**Figure 2:**
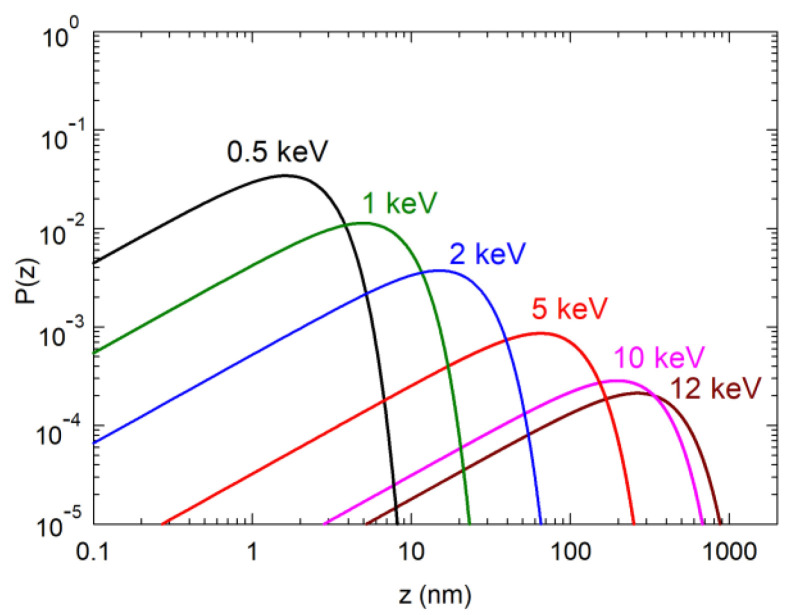
Implantation profiles of positrons with various energies into GaN. Labels indicate positron energy; *z* denotes the depth from the surface.

Positron annihilation spectroscopy (PAS) [13,14] is a well-established technique with high sensitivity to vacancies in solids. Positrons emitted by β+ radioisotopes have a continuous energy spectrum with a mean value in the order of hundreds keV. These energetic positrons are therefore called ‘fast positrons’. The mean penetration depth of fast positrons into solids is in the order of 10^2^–10^3^ μm depending on the material density. Consequently, fast positrons can be used for investigations of bulk samples only. For defect studies of thin films, it is necessary to decrease positron energy. This can be achieved by the moderation of fast positrons. A material with negative positron work function [15] can be used as a moderator. A fraction of fast positrons stopped in the moderator diffuses to its surface and escapes into the vacuum due to the negative positron work function. These so called ‘slow positrons’ are monoenergetic, with an energy of a few eV [16]. Slow positrons are collected in a slow positron beam and guided onto the sample. The energy of slow positrons in the beam can be increased using an electrostatic accelerator. Higher positron energy leads to higher positron penetration depth [16]. Hence, using a variable-energy slow positron beam one can probe the sample from the surface down to various depths. As an example, Figure 2 shows the implantation profiles of positrons with various energies implanted into GaN. The positron implantation profile is well-described by the Makhovian profile [17]. One can see in Figure 2 that varying the energy of slow positrons from 0.5 up to 12 keV enables variation in the mean positron penetration depth into GaN from to 2 to 347 nm. VEPAS is thus well-suited for defect studies of thin films or layered structure. The thickness of GaN films prepared for VEPAS studies is 1 μm (see Figure 1a). Hence, slow positrons are completely stopped inside the GaN layer even for the highest implantation energy of 12 keV used in measurement.

Measurement of a positron’s lifetime requires a start signal providing a time stamp when a positron was born and a stop signal when the positron was annihilated. The stop signal is given by a 511 keV gamma ray emitted during positron annihilation. In order to obtain the start signal, the positron beam has to be pulsed, i.e., positrons are allowed to appear only in narrow time intervals, providing the start signal. The efficiency of positron moderation is very low (≤5 × 10^−3^) [16] and pulsing of the slow positron beam leads to further reduction in intensity. As a consequence, a pulsed variable-energy slow positron beam requires a strong positron source.

VEPAS studies in the present paper were carried out on a LINAC-based pulsed variable energy slow positron beam MePS [18] operating at the ELBE facility [19] at Helmholtz-Zentrum Dresden-Rossendorf. Primary fast positrons were created by pair production induced by electron bremsstrahlung radiation. A tungsten moderator was used to obtain slow positrons. A high-intensity LINAC electron beam (average electron beam current 1.6 mA, electron energy 35 MeV) enabled achieving a high intensity of fast positrons necessary for a pulsed slow positron beam. Moreover, narrow electron pulses of ELBE LINAC (width smaller than 10 ps) were used as start signals for precise measurement of positron lifetimes. The annihilation gamma rays providing stop signals were measured using a CeBr_3_ scintillation detector with digital signal processing. The time resolution of the spectrometer (FWHM of the resolution function) was 230 ps. The energy of slow positrons could be varied in the range from 0.5 to 12 keV.

Photoluminescence (PL) spectra of samples were collected using 325 nm excitation wavelength and a confocal microscope (LabRAM HR Evolution HORIBA, He–Cd laser, objective 74 CG, detector Synapse UV).

Two samples were also prepared for secondary ion mass spectroscopy (SIMS) measurement to study the connection between the contamination of layers and the technology of their preparation (see Figure 1b). These samples contained GaN layers with relevant technological parameters, with a thickness of 50 nm separated by 10 nm thick AlGaN layers that served as markers. All examined layers of the first and second sample were grown in H_2_ and N_2_ atmospheres, respectively. The concentrations of common contaminants (C, O, H, Si) were measured in EAG laboratories [20].

## 3. Results and Discussion

### 3.1. Variable Energy Positron Annihilation Spectroscopy

**Figure 3:**
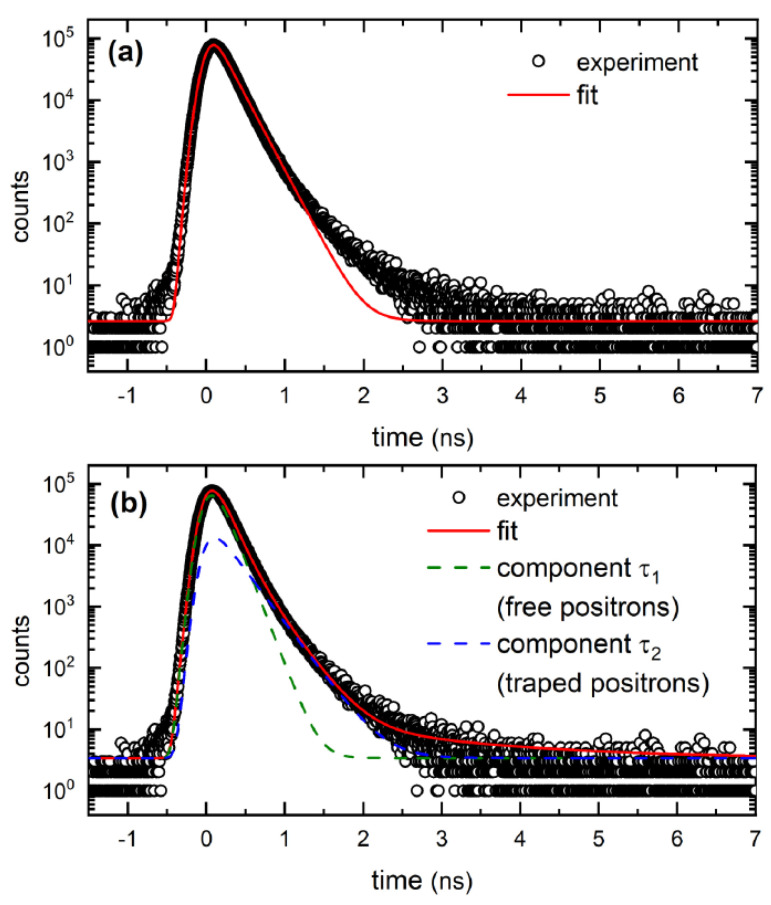
An example of positron lifetime spectrum measured for the TEH1 sample and energy of incident positrons E = 10 keV. Solid line shows fit of the spectrum by model function. (**a**) Single-component fit yielding the mean positron lifetime; (**b**) Two-component fit considering contribution of free positrons (lifetime τ_1_) and positrons trapped at defects (lifetime τ_2_). Individual components are plotted by dashed lines.

**Figure 4:**
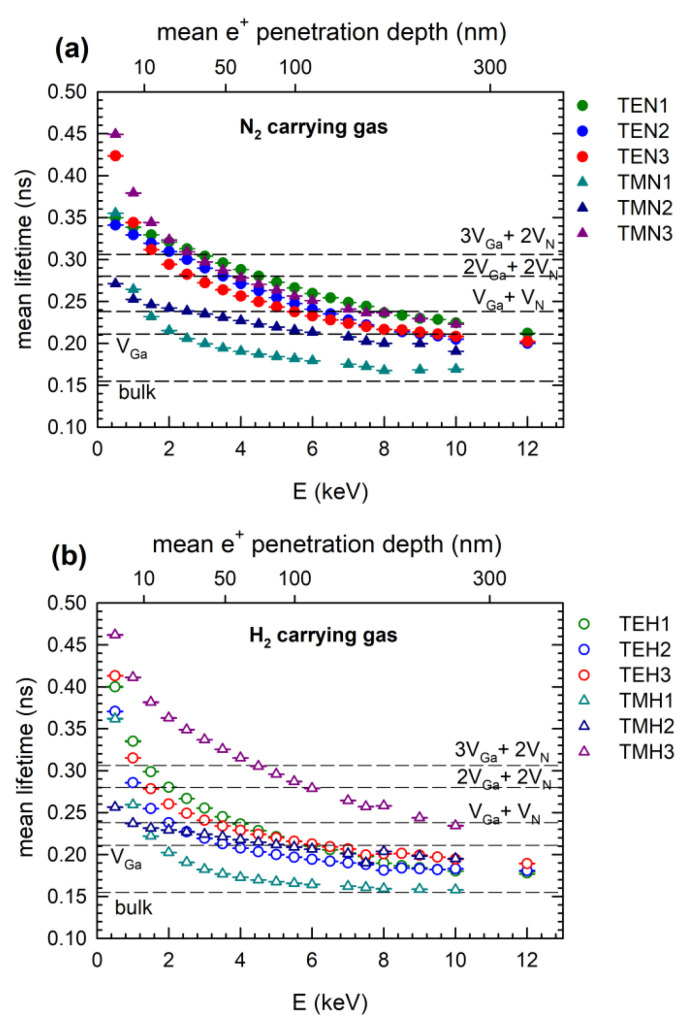
Dependence of the mean positron lifetime on the energy of incident positrons entering the GaN layer prepared under different growth conditions in (**a**) nitrogen and (**b**) hydrogen atmosphere. Upper *x*-axis shows the mean positron penetration depth.

A positron lifetime spectrum is in general a sum of exponential components convoluted with the response function of the spectrometer. Each component corresponds to a certain positron state and is characterized by its lifetime *τ_i_* and relative intensity *I_i_*. A single-component fit of positron lifetime spectra yields the mean positron lifetime, representing the weighted average of lifetimes of various components, $\bar{\tau }=\overset{}{\underset{i}{\sum }}\tau_{i}I_{i}.$ An example of a single-component fit of positron lifetime spectrum for the TEH1 sample for positron energy E = 10 keV is shown in Figure 3a. The mean positron lifetime is a robust parameter not affected by mutual correlations among fitting parameters and provides useful information about trends in the series of GaN films studied. The mean positron lifetime for GaN layers studied is plotted in Figure 4 as a function of the positron energy. At low energies, almost all positrons are annihilated on the surface and the mean positron lifetime corresponds to the surface state. With increasing energy, positrons penetrate deeper into the GaN layer and the fraction of positrons diffusing back to the surface gradually decreases. Consequently, the mean positron lifetime gradually decreases with increasing positron energy from the surface value down to a value corresponding to when all positrons are annihilated in the GaN layer.

Positrons are attracted and captured by negatively charged Ga vacancies (V_Ga_). In the empty volume of a V_Ga_, positrons have lower overlap with the electrons of crystal atoms, which decreases the probability of their annihilation and, hence, the lifetime of positrons trapped in the vacancy is increased. Figure 4 also shows the calculated lifetime values of free positrons in a perfect GaN crystal without defects (bulk) as well as that of positrons trapped in V_Ga_ and of positrons trapped in different clusters with nitrogen vacancies (V_N_). Positron lifetime increased with increased empty volume in the defect. Positrons annihilated on the surface influenced the measured mean lifetime values up to the mean penetration depth of 200–300 nm due to the back-diffusion of positrons toward the surface. For higher penetration depths, i.e., incident positron energy above 8 keV, the fraction of positrons diffusing back to the surface becomes negligible and almost all positrons are annihilated in the GaN layer. The mean positron lifetime values for energies above 8 keV were, therefore, supposed to be relevant for determination of defect type and concentration.

From inspection of Figure 4, one can conclude that the mean positron lifetime at high energies (E > 8 keV) approaches values located between the calculated bulk positron lifetime (i.e., lifetime of free positrons in a defect-free lattice) and the calculated lifetime of positrons trapped in V_Ga_. This indicates that GaN films contain V_Ga_, and some positrons are trapped and annihilated in V_Ga_ while the remaining positrons are annihilated in the free state, i.e., not trapped at defects. The only exception comprises TMN3 and TMH3 samples grown at the highest temperature of 1100 °C. In TMN3 and TMH3, the mean positron lifetime at high energies approached a value that was higher than the calculated lifetime for V_Ga_. This indicates that TMN3 and TMH3 contain defects with a higher open volume than V_Ga_.

**Figure 5:**
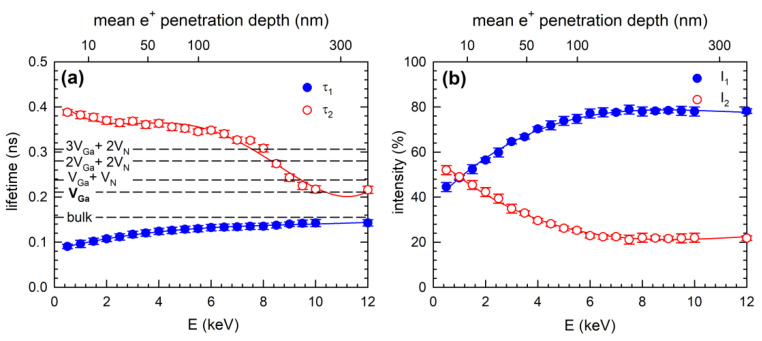
Breakdown results of positron lifetime spectra for sample TEH1 (**a**) lifetimes τ_1_ and τ_2_ of components resolved in spectra and (**b**) intensities I_1_ and I_2_ of the components. Dashed lines indicate calculated bulk positron lifetime and lifetimes of positrons trapped in various defects. The mean positron penetration depth is shown in the upper *x*-axis. The solid lines serve to guide the eye.

**Figure 6:**
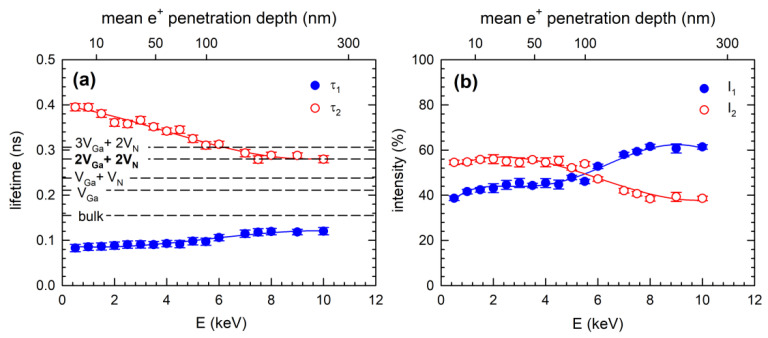
Results of breaking down positron lifetime spectra for the TMH3 sample (**a**) lifetimes τ_1_ and τ_2_ of the components resolved in spectra and (**b**) intensities I_1_ and I_2_ of the components. Dashed lines indicate calculated bulk positron lifetime and the lifetimes of positrons trapped in various defects. The mean positron penetration depth is shown in the upper *x*-axis. The solid lines serve to guide the eye.

**Figure 7:**
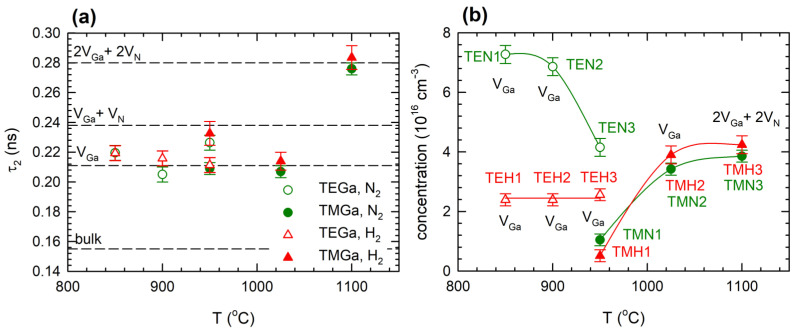
(**a**) The development of the lifetime τ_2_ measured for the energy of incident positrons of 12 keV on different film deposition temperatures; (**b**) the concentration of V_Ga_ determined by VEPAS for samples prepared with growth parameters defined in Table 1. Labels indicate notation of sample and dominant type of defects in the sample. Solid lines are plotted to guide eyes only.

An example of the decomposition of the positron lifetime spectra for the TEH1 sample and the energy of incident positrons E = 10 keV is shown in Figure 3b. From comparison of Figure 3a to Figure 3b it is clear that fitting by two components results in better agreement with experimental points than the single-component fit. The development of positron lifetimes τ_1_ and τ_2_ and corresponding intensities I_1_ and I_2_ for sample TEH1 with positron energy is shown in Figure 5a and Figure 5b, respectively. It can be noticed that for penetration depth 300 nm, the positron lifetime τ_2_ reached the value calculated for positrons trapped in V_Ga_, testifying that the TEH1 sample contains V_Ga_. Results of the breakdown of positron lifetime spectra for other samples are collected in Supplementary Materials. Similar results as for TEH1 were also obtained for other GaN films deposited at temperatures below 1100 °C. The results of the decomposition of positron lifetime spectra for the TMH3 sample deposited at 1100 °C are plotted in Figure 6. In contrast to the films deposited at lower temperatures, the lifetime τ_2_ for the TMH3 sample at high energies approaches a significantly higher value, corresponding to the calculated lifetime of positrons trapped in a complex consisting of two Ga and two N missing ions (2V_Ga_+2V_N_ complex). A similar result was obtained for TMN3 film deposited at 1100 °C using N_2_ carrying gas (see Supplementary Materials). The development of the lifetime τ_2_ measured at the highest positron energy of 12 keV on different GaN film deposition temperatures is plotted in Figure 7a. For deposition temperatures lower than 1100 °C, the lifetime τ_2_ remains close to the calculated value for V_Ga_. However, for films deposited at 1100 °C the lifetime τ_2_ increases to the value corresponding to a 2V_Ga_ + 2V_N_ complex. Thus, one can conclude that GaN films grown at temperatures below 1100 °C contain V_Ga_. However, at higher temperatures the nature of the defects changes and films grown at 1100 °C contain 2V_Ga_ + 2V_N_ complexes.

The V_Ga_ concentration was calculated from the lifetimes and intensities of both components using the two-state trapping model [21]. Data measured for the highest positron energy of 12 keV corresponding to the highest positron penetration depth were used for determination of the vacancy concentration for all samples. Results for all samples are summarized in Figure 7b. V_Ga_ formation is not only influenced by the temperature, but it is also strongly dependent on the choices of precursor and carrier gas. The temperature dependence of V_Ga_ concentration for samples grown from TEGa and TMGa precursors have opposite characters. Unfortunately, there was not sufficient overlap of temperature intervals in which both precursors could be used for epitaxy; only around 950 °C could both types of precursors be used. TEGa is preferred for temperatures below 950 °C, since at this temperature range TMGa is less decomposed and too much carbon would be incorporated into the layers. At temperatures above 950 °C, TEGa cannot be used due to adduct formation, which impacts the growth rate.

Very characteristic of layers grown from TEGa is that the measured V_Ga_ concentration is significantly higher in a nitrogen atmosphere compared to samples grown in hydrogen. In the case of TEGa grown in nitrogen, V_Ga_ concentration decreases with temperature. This is unexpected from a thermodynamic point of view, since the vacancy concentration should be increasing with temperature as exp (-E_f_/kT), where E_f_ is the vacancy formation energy [22]. Several possible hypotheses explaining this behaviour can be suggested. Since the TEGa precursor should be fully decomposed in the studied temperature range [23], limited Ga precursor decomposition can be ruled out. One of the possible explanations is the influence of surface morphology on vacancy formation, because in a N_2_ atmosphere the epitaxial surface is rather rough (V-shape defects are formed). A lower V_Ga_ concentration in a H_2_ atmosphere can be due to passivation or filling of V_Ga_ by hydrogen incorporated in the GaN lattice. In complexes of V_Ga_ associated with H atoms positrons cannot be trapped or their lifetime is much shorter. For comparison, the lifetime of a positron trapped in a V_Ga_ is 200–211 ps, while positron lifetime for V_Ga_-H complexes is shortened to 180 ps and for V_Ga_-2H it is further shortened to 150 ps, so it is even shorter than 155 ps, which is the bulk positron lifetime for GaN, i.e., lifetime of free positrons in a perfect (defect-free) GaN crystal [24].

When TMGa is used as the precursor, V_Ga_ concentration is not influenced by the choice of atmosphere in the MOVPE reactor, but it is strongly enhanced with increasing temperature. The lifetime corresponding to a cluster 2V_Ga_–2V_N_ was measured for the highest growth temperature of 1100 °C, which means that the actual V_Ga_ concentration was twice the concentration of 2V_Ga_–2V_N_ complexes shown in Figure 7b, since each complex contains two V_Ga_. Significantly different V_Ga_ concentration when TMGa or TEGa was used for the GaN growth suggests that the reaction pathway has important influence on the vacancy formation. This could be a reason why theoretical predictions of V_Ga_ concentration based on formation energy [25] are underestimated. In the future, layers grown from novel precursors and the vacancy formation in them might help to understand the growth kinetics, as suggested in [26,27].

### 3.2. Photoluminescence

Knowing the V_Ga_ concentration in particular GaN layers, we investigated its correlation with PL properties. It could bring valuable insight into the quest for yellow band (YB) luminescence origin. In previous works, YB luminescence was attributed to a C_N_ defect [28], C_N_-O_N_ complex [29], which is energetically favourable, or C_N_-Si_Ga_ [30] in n-type GaN layers. Other sources of YB luminescence that were proposed in the literature were V_Ga_-3H and V_Ga_-O_N_-2H complexes [31] or V_Ga_-O_N_ [32]. V_Ga_ were also supposed to be responsible for nonradiative recombination [6].

**Figure 8:**
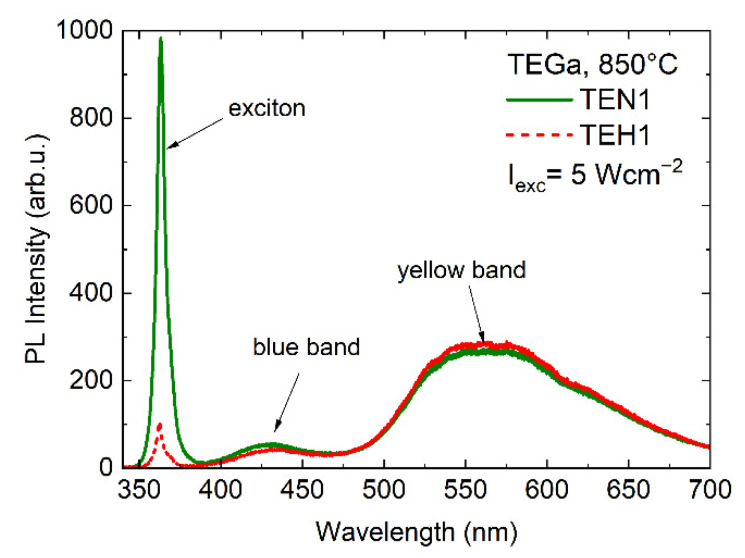
Room-temperature photoluminescence spectra of samples TEN1 and TEH1.

Typical room-temperature PL spectra for MOVPE-prepared GaN layers (samples TEN1 and TEH1) are shown in Figure 8 to illustrate the position of PL bands. Contrary to expectations, for sample TEN1, with a higher V_Ga_ concentration, neither an increase in YB intensity nor a decrease of PL efficiency was observed.

**Figure 9:**
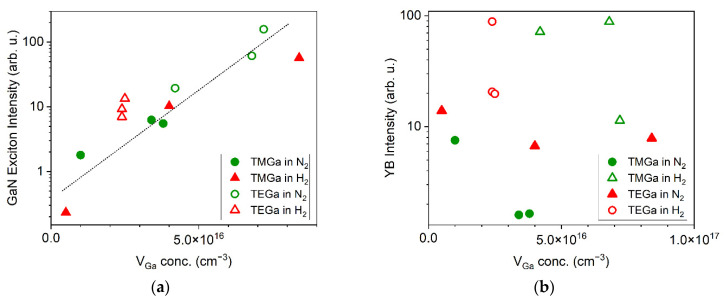
Maximum intensity of (**a**) excitonic and (**b**) yellow band luminescence as a function of V_Ga_ concentration.

In Figure 9a,b, correlation between excitonic and YB intensity with respect to the V_Ga_ concentration is shown, summarizing the data obtained from all studied layers. No correlation between V_Ga_ concentration and YB luminescence intensity was observed, which suggests that V_Ga_ are not the dominant reason for YB luminescence.

However, there is unexpected and clear positive correlation between V_Ga_ concentration and excitonic luminescence. It means that V_Ga_ cannot be efficient nonradiative centre. Since there is no known mechanism in which V_Ga_ presence would enhance free-exciton emission in GaN, we suggest the following explanation for the correlation observed in Figure 9a: higher exciton intensity is caused by the suppression of other recombination channels, especially the nonradiative ones (because enhancement of yellow luminescence was not observed with increasing V_Ga_ concentration, Figure 9b). Correlation between V_Ga_ concentration and GaN exciton intensity means that there exists an anticorrelation between nonradiative centre concentration and V_Ga_ concentration. In other words, the formation of an unknown nonradiative centrum is suppressed when the Ga vacancies are present.

### 3.3. Secondary Ion Mass Spectroscopy

**Figure 10:**
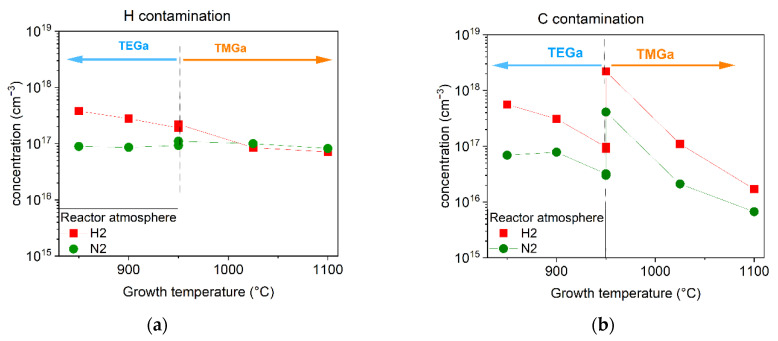
Concentration of (**a**) hydrogen and (**b**) carbon contamination measured by SIMS on layers prepared on multi-layered samples with technological parameters as defined in Table 1.

To find possible candidates for nonradiative defects that are anticorrelated with V_Ga_ and to elucidate whether V_Ga_ form complexes with atoms from impurities, we prepared multilayer samples designed for SIMS characterization according to the scheme in Figure 1b. Hydrogen, oxygen, carbon, and silicon concentration profiles were measured by SIMS. There was one significant feature observed in all GaN layers—contamination from all four elements was higher when H_2_ was used for the reactor atmosphere. Interesting correlations between V_Ga_ and carbon for samples grown from TMGa, and between V_Ga_ and hydrogen for samples grown from TEGa were observed. The obtained hydrogen and carbon concentrations for GaN layers prepared according to the above-defined technological parameters are summarized in Figure 10a,b.

In layers grown in a H_2_ atmosphere at lower temperatures, hydrogen contamination was approximately three times higher than for samples grown in a N_2_ atmosphere. We suppose that NH_3_ is less efficiently decomposed in a H_2_ atmosphere and hydrogen could be incorporated into the epitaxial layer. Hydrogen can form complexes with V_Ga_ that are invisible to PAS methods, as was explained above. Since in samples grown from TEGa in a H_2_ atmosphere the oxygen concentration measured by SIMS was in the order of 10^17^ cm^−3^ (not shown here), we can also suppose the formation of V_Ga_-O_N_-2H, which is energetically favourable. In fact, if H and O are abundant, formation of V_Ga_-3H, V_Ga_-2H, or V_Ga_-O_N_-2H complexes is much more probable than formation of isolated V_Ga_. The smallest formation energy of V_Ga_ is found when the Fermi level is near the conduction band minimum but still reaches a value around 4.5 eV. On the other hand, at the same Fermi level position, the formation energy of V_Ga_-3H, V_Ga_-2H, and V_Ga_-O_N_-2H is around 3 eV, 2.6 eV, and 0.5 eV, respectively [33]. Since none of these defects can be detected by VEPAS due to the small open volume of the defects, their presence may explain the significantly lower detected V_Ga_ concentration in layers grown from TEGa in a H_2_ atmosphere. Some of these defects probably also act as nonradiative centres [33].

However, in samples grown from TMGa, hydrogen does not play a significant role. Instead, carbon contamination seems to be anticorrelated with V_Ga_ concentration. It seems that for the lowest temperature of 950 °C, the carbon concentration is so high that it is filling not only V_N_, but also V_Ga_, thus forming n-type C_Ga_ defects. On the other hand, at the highest temperature, when there is low carbon contamination, both types of vacancies remain unfilled and their concentration is so high that they can merge into clusters, as was observed at 1100 °C. According to the PL results, at the highest carbon concentration PL efficiency drops significantly, suggesting the formation of strong nonradiative defects, which could be C_Ga_ according to [34], or it may be due to some complex related to C_Ga_ such as C_Ga_-C_N_ or C_Ga_-H_i_.

## 4. Conclusions

Twelve samples with GaN layers prepared by MOVPE under different technological conditions were investigated by a unique VEPAS method to find a link between technological conditions and V_Ga_ formation. Different correlations between V_Ga_ concentration and technology parameters were observed for layers grown from TEGa and TMGa precursors. In the case of TEGa, V_Ga_ formation was significantly influenced by the type of reactor atmosphere (N_2_ or H_2_), while no similar behaviour was observed for samples grown from TMGa. V_Ga_ formation was suppressed with increasing temperature for growth from TEGa in a N_2_ atmosphere. On the contrary, opposite temperature dependence for V_Ga_ formation was observed for growth from TMGa, with cluster formation for the highest temperature of 1100 °C. Significantly different V_Ga_ formation when TMGa or TEGa was used for the GaN growth suggests that the reaction mechanism has an important influence on formation of vacancies. This could be a reason why V_Ga_ concentrations theoretically predicted from formation energies are underestimated. According to the correlation of PL results with V_Ga_ concentrations determined by VEPAS, it can be concluded that YB luminescence is not generally connected with V_Ga_ in MOVPE-prepared GaN layers. Surprisingly, the presence of V_Ga_ was correlated with intensity of excitonic luminescence. The probable explanation is that the V_Ga_ concentration in GaN layers is anticorrelated with some efficient nonradiative defects. According to SIMS results and correlation with detected V_Ga_ concentrations, we have suggested several candidates for such nonradiative defects, which could be C_Ga,_ C_Ga_-C_N_, or C_Ga_ -H_i_ in samples grown from TMGa and V_Ga_-3H, V_Ga_-2H, or V_Ga_-O_N_-2H complexes in samples grown from TEGa.

## Figures and Tables

**Table 1:** Technological growth conditions of samples prepared for VEPAS measurement.

Samples		Temperature	Precursor
N_2_ atmosphere	H_2_ atmosphere		
TEN1	TEH1	850 °C	TEGa
TEN2	TEH2	900 °C	TEGa
TEN3	TEH3	950 °C	TEGa
TMN1	TMH1	950 °C	TMGa
TMN2	TMH2	1025 °C	TMGa
TMN3	TMH3	1100 °C	TMGa

## Data Availability

Measured data can be provided upon request from hospodko@fzu.cz.

## Supplementary Materials

The following supporting information can be downloaded at: https://www.mdpi.com/article/10.3390/ma15196916/s1, Figure S1: Results of decompositions of positron lifetime spectra for the sample TEN1 plotted as a function of the energy of incident positrons. Figure S2: Results of decompositions of positron lifetime spectra for the sample TEN2 plotted as a function of the energy of incident positrons. Figure S3: Results of decompositions of positron lifetime spectra for the sample TEN3 plotted as a function of the energy of incident positrons. Figure S4: Results of decompositions of positron lifetime spectra for the sample TEH1 plotted as a function of the energy of incident positrons. Figure S5: Results of decompositions of positron lifetime spectra for the sample TEH2 plotted as a function of the energy of incident positrons. Figure S6: Results of decompositions of positron lifetime spectra for the sample TEH3 plotted as a function of the energy of incident positrons. Figure S7: Results of decompositions of positron lifetime spectra for the sample TMN1 plotted as a function of the energy of incident positrons. Figure S8: Results of decompositions of positron lifetime spectra for the sample TMN2 plotted as a function of the energy of incident positrons. Figure S9: Results of decompositions of positron lifetime spectra for the sample TMN3 plotted as a function of the energy of incident positrons. Figure S10: Results of decompositions of positron lifetime spectra for the sample TMH1 plotted as a function of the energy of incident positrons. Figure S11: Results of decompositions of positron lifetime spectra for the sample TMH2 plotted as a function of the energy of incident positrons. Figure S12: Results of decompositions of positron lifetime spectra for the sample TMH3 plotted as a function of the energy of incident positrons. References [35,36] are cited in the supplementary materials.
