# Supplementary material for: Relation between Ga Vacancies, Photoluminescence, and Growth Conditions of MOVPE-Prepared GaN Layers
Source: Materials (Basel). 2022 Oct 5;15(19):6916. doi: 10.3390/ma15196916 (PMC9572910; doi:10.3390/ma15196916)
Supplement: Supplementary file 1 [file materials-15-06916-s001.zip › materials-1921081-supplementary.pdf]

materials

Supplementary

Relation between Ga vacancies, photoluminescence and growth conditions of MOVPE prepared GaN layers

Alice Hospodková [1,*], Jakub Čížek [2], František Hájek [1,3], Tomáš Hubáček [1], Jiří Pangrác [1], Filip Dominec [1], Karla Kuldová [1], Jan Batysta [1,3], Maciej Oskar Liedke [4], Eric Hirschmann [4], Maik Butterling [4] and Andreas Wagner [4]

[1] Institute of Physics CAS, Cukrovarnická 10, 162 00 Prague, Czech Republic
[2] Faculty of Mathematics and Physics, Charles University, V Holešovičkách 2, 180 00 Prague, Czech Republic
[3] Faculty of Nuclear Sciences and Physical Engineering, Czech Technical University, Břehová 7, 115 19, Prague, Czech Republic
[4] Institute of Radiation Physics, Helmholtz-Zentrum Dresden-Rossendorf Bautzner Landstr. 400, 01328 Dresden, Germany
* Correspondence: hospodko@fzu.cz

A—decomposition of positron lifetime spectra

Positron lifetime spectrum is a sum of exponential components convoluted with the resolution function of the spectrometer and a constant background originating from random coincidences. In the present case the resolution function was described as a sum of two Gaussians shifted with respect to each other. Positron lifetime spectra measured for GaN films were fitted using a PLRF code [1]. Each component is characterized by its lifetime τ_i and relative intensity I_i and corresponds to certain positron state in the sample.

Three components were assumed in decomposition of positron lifetime spectra of GaN films: (i) a short-lived component with lifetime τ_1 comes from positrons annihilated in the free state (not trapped at defects); (ii) a component with lifetime τ_2 represents a contribution of positrons annihilated in the surface state or trapped at defects (V_{Ga} or $2V_{Ga}$–$2V_N$) in GaN layer; (iii) a weak long-lived component with lifetime τ_3 can be attributed to pick-off annihilation of ortho-positronium (o-Ps) [2] formed on the sample surface. The development of lifetimes and relative intensities of all components resolved in spectra on the energy of incident positrons for all GaN samples studied is plotted in Figures S1-S12. Upper panels in each figure always show the development of the components (i) and (ii) from free positrons and positrons trapped at defects while lower panels present the behaviour of the o-Ps contribution. It is clear that the intensity of the o-Ps quickly diminishes with increasing positron energy testifying that o-Ps is formed on the surface only.

At low energies almost all positrons are annihilated on the surface. With increasing energy positrons penetrate deeper into GaN layer and the fraction of positrons diffusing back to the surface gradually decreases. It is reflected by a decrease of the lifetime τ_2 from a surface value of 0.3-0.4 ns to a value corresponding to the situation when all positrons are annihilated inside the GaN layer. The latter value agrees well with the calculated lifetime of positrons trapped in V_{Ga} for all films deposited below 1100°C. In the case of samples TMN3 (Figure S9) and TMH3 (Figure S12) grown at 1100°C the lifetime τ_2 is remarkably higher and approaches at high energies the value calculated for positrons trapped at $2V_{Ga}$ + $2V_N$ complexes. The intensity I_2 gradually decreases with increasing positron energy reflecting decreasing contribution of positrons annihilated on the surface. The intensity I_1 of the free positron component increases with positron energy due to increasing fraction of positorns annihilated inside GaN layer and the lifetime τ_1 gradually increases towards the bulk value.

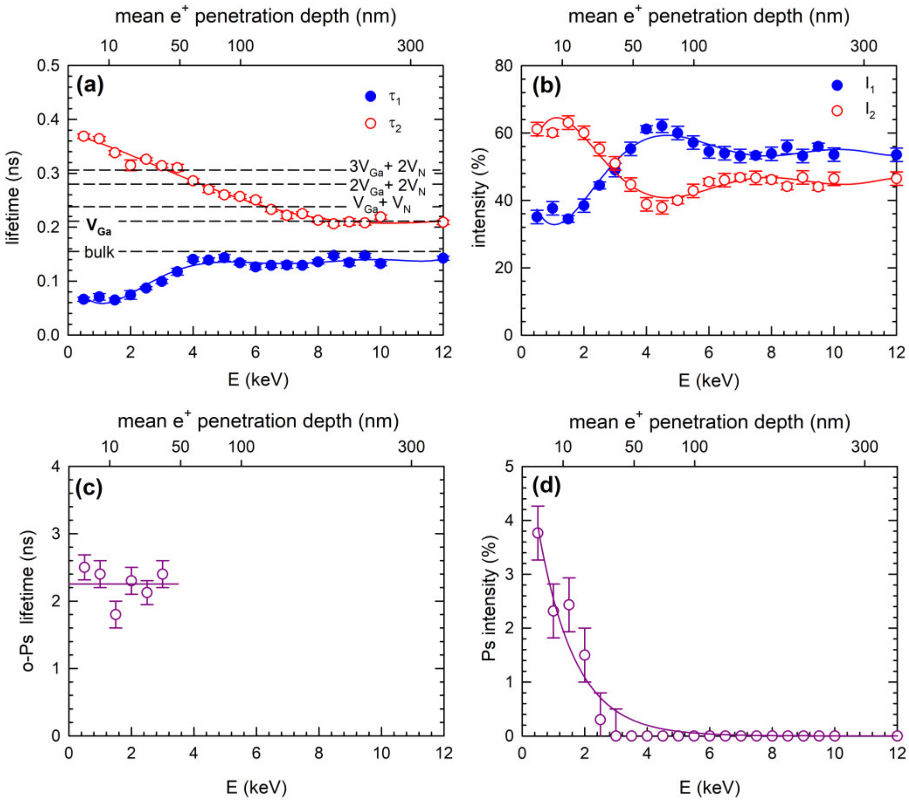

Figure S1. Results of decompositions of positron lifetime spectra for the sample TEN1 plotted as a function of the energy of incident positrons. (a) the development of lifetimes τ_1 and τ_2 of positrons annihilated in the free state and trapped at defects, respectively; (b) corresponding intensities I_1, I_2; (c) lifetime of o-Ps pick-off annihilation; (d) intensity of the o-Ps contribution. The mean positron penetration depth is shown in the upper x-axis. Dashed lines in the panel (a) indicate calculated lifetime of free positrons in perfect GaN crystal (bulk) and positrons trapped at various defects.

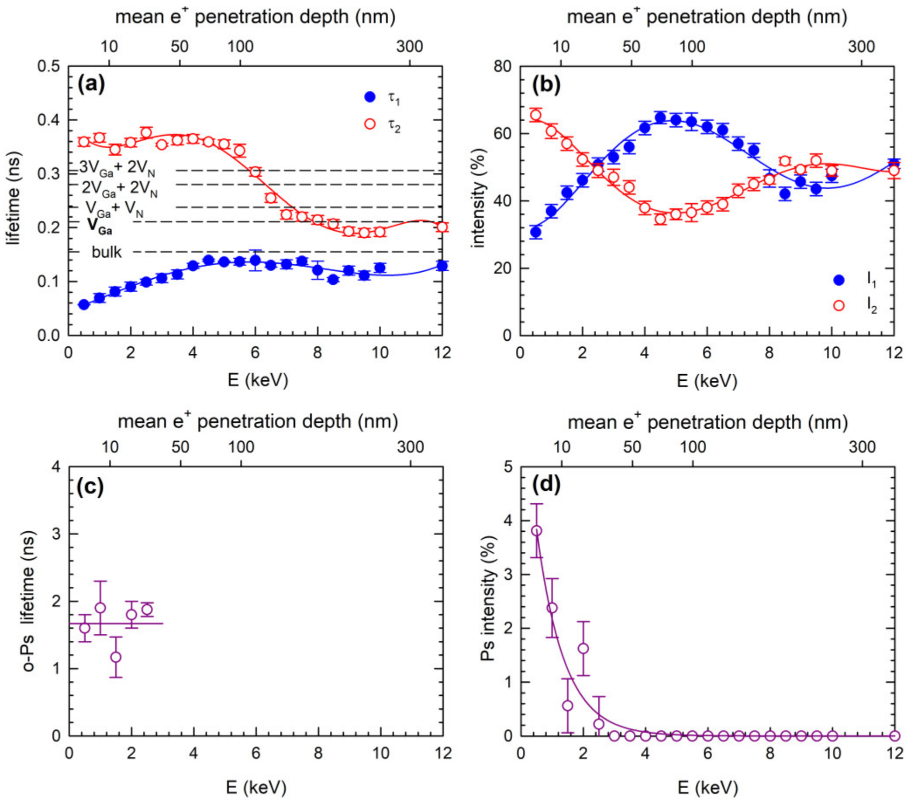

Figure S2. Results of decompositions of positron lifetime spectra for the sample TEN2 plotted as a function of the energy of incident positrons. (a) the development of lifetimes τ_1 and τ_2 of positrons annihilated in the free state and trapped at defects, respectively; (b) corresponding intensities I_1, I_2; (c) lifetime of o-Ps pick-off annihilation; (d) intensity of the o-Ps contribution. The mean positron penetration depth is shown in the upper x-axis. Dashed lines in the panel (a) indicate calculated lifetime of free positrons in perfect GaN crystal (bulk) and positrons trapped at various defects.

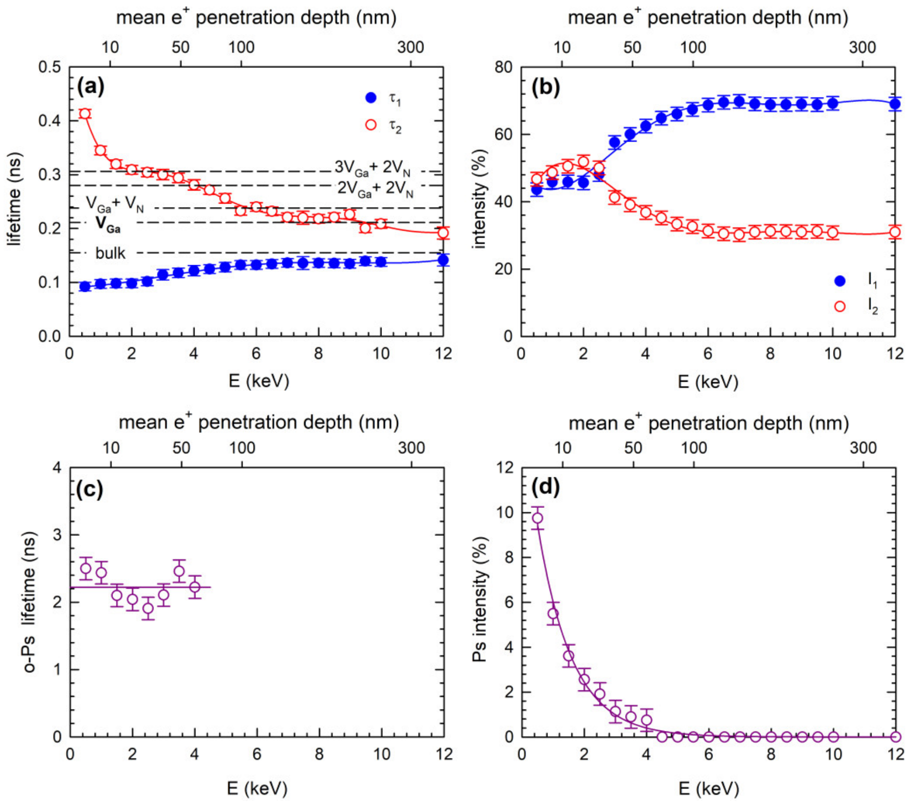

Figure S3. Results of decompositions of positron lifetime spectra for the sample TEN3 plotted as a function of the energy of incident positrons. (a) the development of lifetimes τ_1 and τ_2 of positrons annihilated in the free state and trapped at defects, respectively; (b) corresponding intensities I_1, I_2; (c) lifetime of o-Ps pick-off annihilation; (d) intensity of the o-Ps contribution. The mean positron penetration depth is shown in the upper x-axis. Dashed lines in the panel (a) indicate calculated lifetime of free positrons in perfect GaN crystal (bulk) and positrons trapped at various defects.

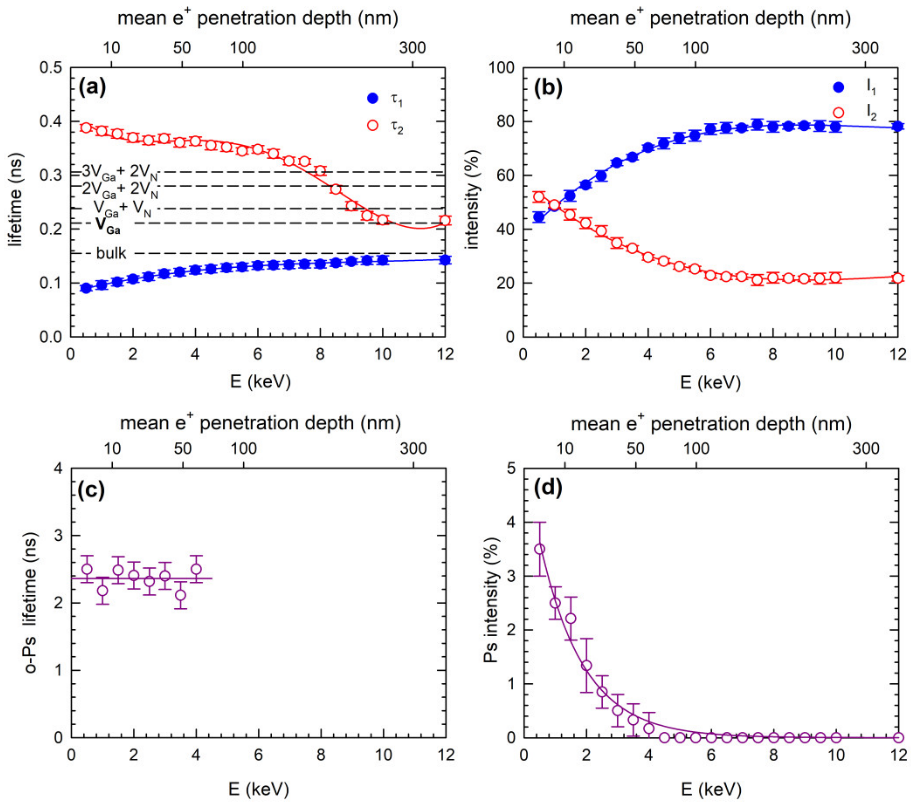

Figure S4. Results of decompositions of positron lifetime spectra for the sample TEH1 plotted as a function of the energy of incident positrons. (a) the development of lifetimes τ_1 and τ_2 of positrons annihilated in the free state and trapped at defects, respectively; (b) corresponding intensities I_1, I_2; (c) lifetime of o-Ps pick-off annihilation; (d) intensity of the o-Ps contribution. The mean positron penetration depth is shown in the upper x-axis. Dashed lines in the panel (a) indicate calculated lifetime of free positrons in perfect GaN crystal (bulk) and positrons trapped at various defects.

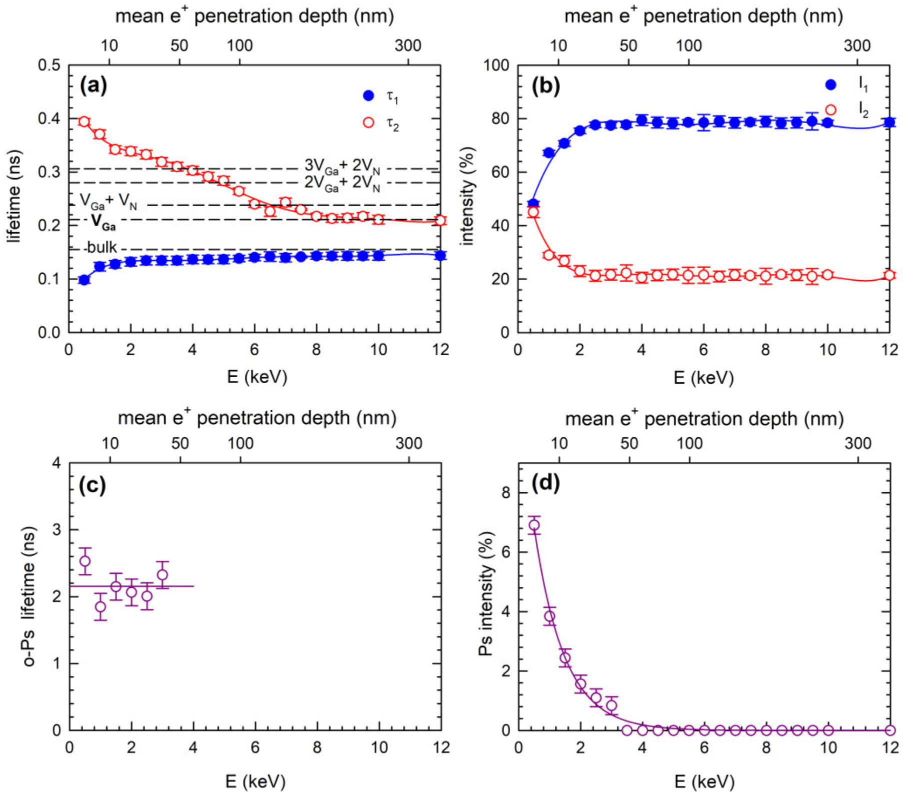

Figure S5. Results of decompositions of positron lifetime spectra for the sample TEH2 plotted as a function of the energy of incident positrons. (a) the development of lifetimes τ_1 and τ_2 of positrons annihilated in the free state and trapped at defects, respectively; (b) corresponding intensities I_1, I_2; (c) lifetime of o-Ps pick-off annihilation; (d) intensity of the o-Ps contribution. The mean positron penetration depth is shown in the upper x-axis. Dashed lines in the panel (a) indicate calculated lifetime of free positrons in perfect GaN crystal (bulk) and positrons trapped at various defects.

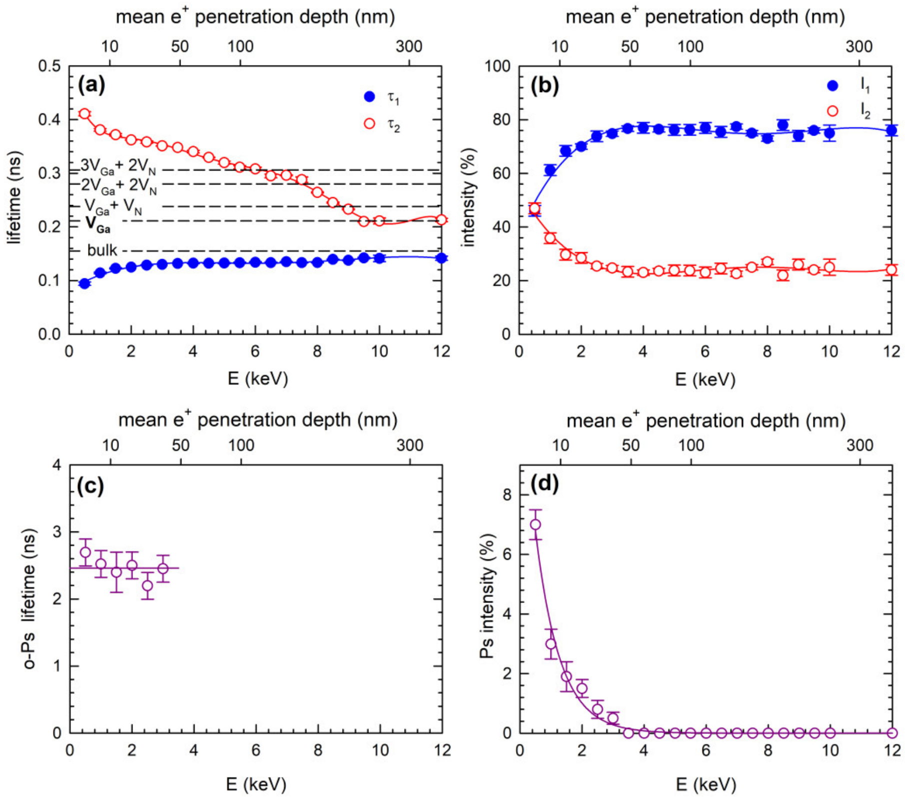

Figure S6. Results of decompositions of positron lifetime spectra for the sample TEH3 plotted as a function of the energy of incident positrons. (a) the development of lifetimes τ_1 and τ_2 of positrons annihilated in the free state and trapped at defects, respectively; (b) corresponding intensities I_1, I_2; (c) lifetime of o-Ps pick-off annihilation; (d) intensity of the o-Ps contribution. The mean positron penetration depth is shown in the upper x-axis. Dashed lines in the panel (a) indicate calculated lifetime of free positrons in perfect GaN crystal (bulk) and positrons trapped at various defects.

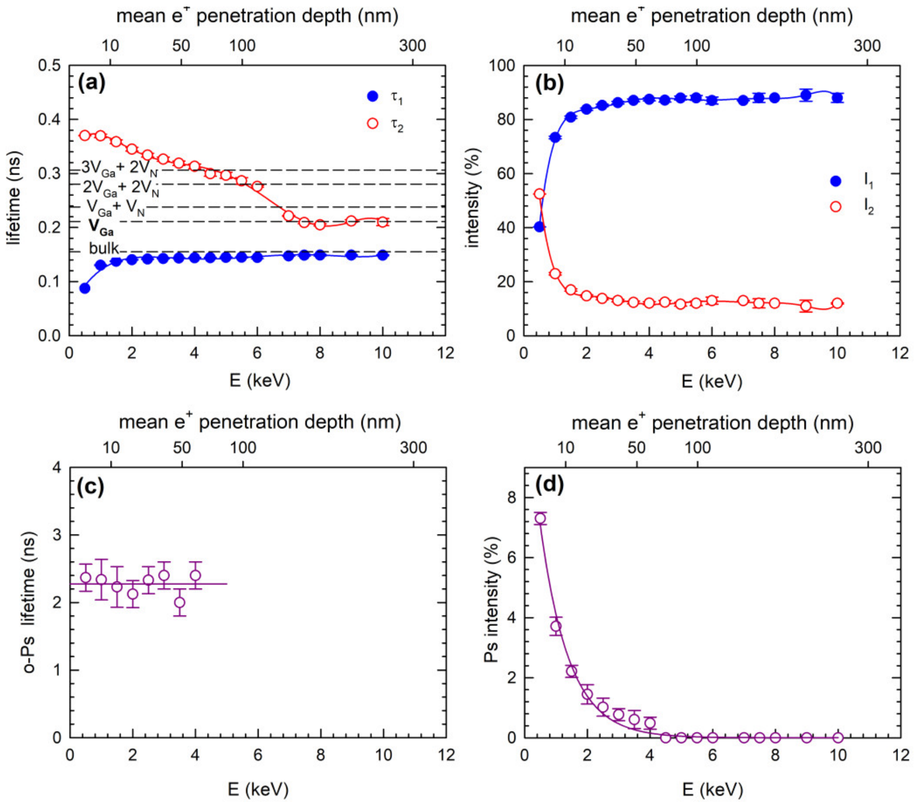

Figure S7. Results of decompositions of positron lifetime spectra for the sample TMN1 plotted as a function of the energy of incident positrons. (a) the development of lifetimes τ_1 and τ_2 of positrons annihilated in the free state and trapped at defects, respectively; (b) corresponding intensities I_1, I_2; (c) lifetime of o-Ps pick-off annihilation; (d) intensity of the o-Ps contribution. The mean positron penetration depth is shown in the upper x-axis. Dashed lines in the panel (a) indicate calculated lifetime of free positrons in perfect GaN crystal (bulk) and positrons trapped at various defects.

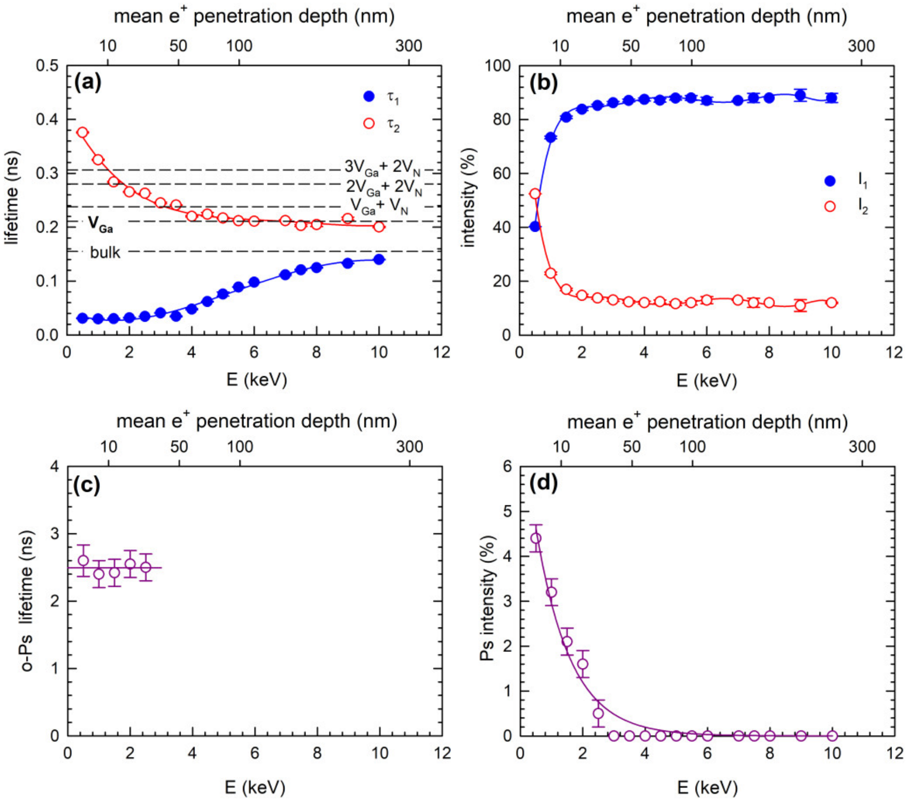

Figure S8. Results of decompositions of positron lifetime spectra for the sample TMN2 plotted as a function of the energy of incident positrons. (a) the development of lifetimes τ_1 and τ_2 of positrons annihilated in the free state and trapped at defects, respectively; (b) corresponding intensities I_1, I_2; (c) lifetime of o-Ps pick-off annihilation; (d) intensity of the o-Ps contribution. The mean positron penetration depth is shown in the upper x-axis. Dashed lines in the panel (a) indicate calculated lifetime of free positrons in perfect GaN crystal (bulk) and positrons trapped at various defects.

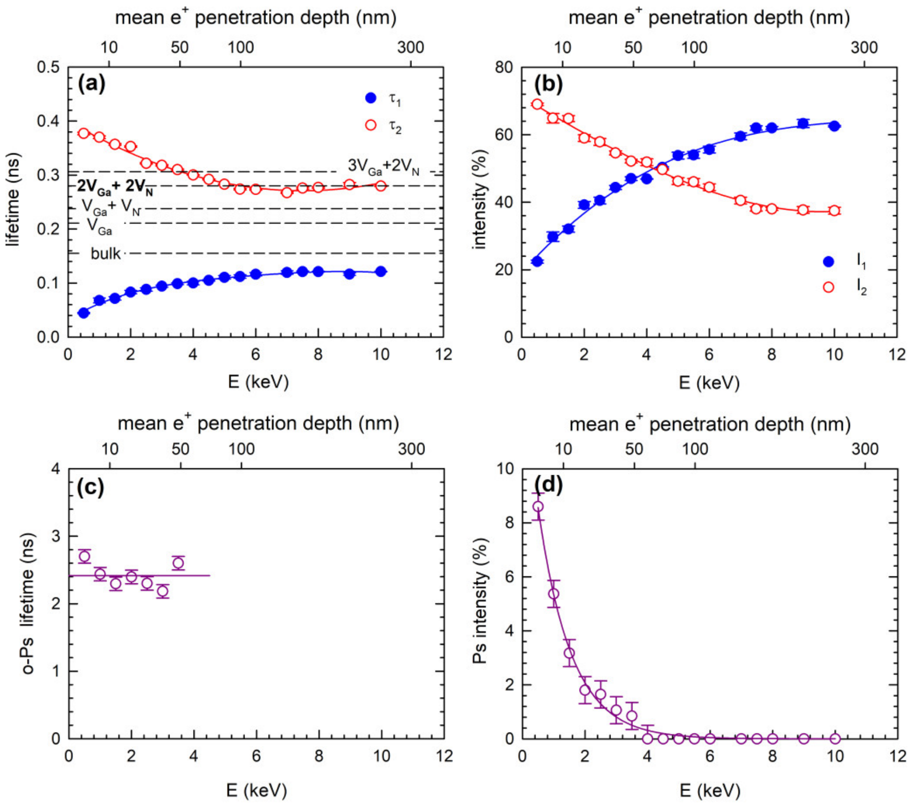

Figure S9. Results of decompositions of positron lifetime spectra for the sample TMN3 plotted as a function of the energy of incident positrons. (a) the development of lifetimes τ_1 and τ_2 of positrons annihilated in the free state and trapped at defects, respectively; (b) corresponding intensities I_1, I_2; (c) lifetime of o-Ps pick-off annihilation; (d) intensity of the o-Ps contribution. The mean positron penetration depth is shown in the upper x-axis. Dashed lines in the panel (a) indicate calculated lifetime of free positrons in perfect GaN crystal (bulk) and positrons trapped at various defects.

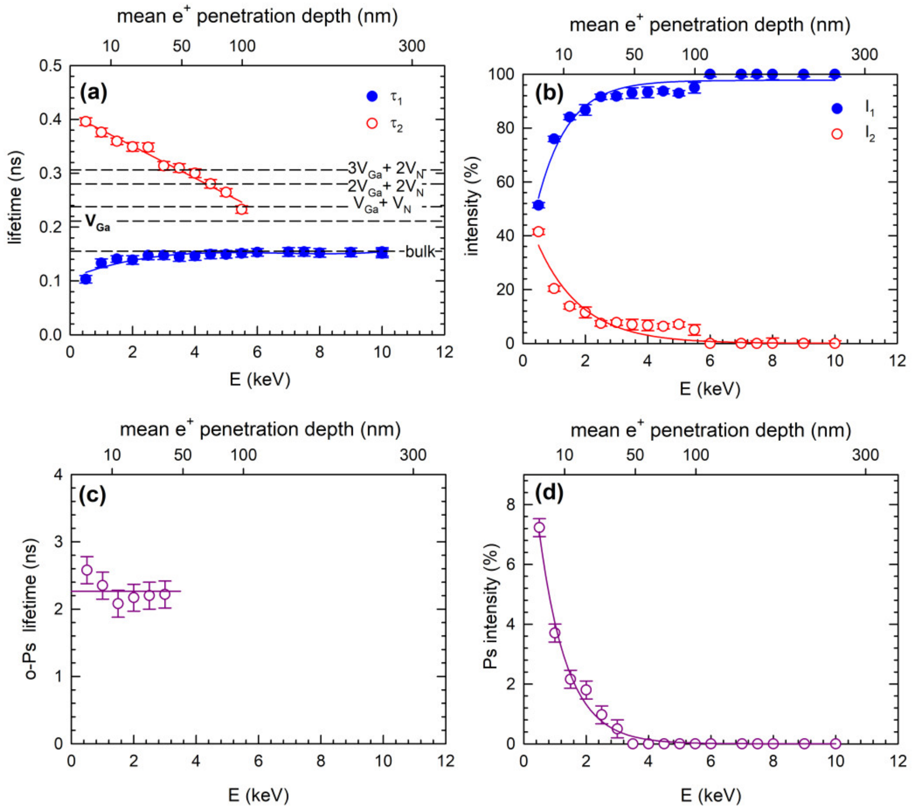

Figure S10. Results of decompositions of positron lifetime spectra for the sample TMH1 plotted as a function of the energy of incident positrons. (a) the development of lifetimes τ_1 and τ_2 of positrons annihilated in the free state and trapped at defects, respectively; (b) corresponding intensities I_1, I_2; (c) lifetime of o-Ps pick-off annihilation; (d) intensity of the o-Ps contribution. The mean positron penetration depth is shown in the upper x-axis. Dashed lines in the panel (a) indicate calculated lifetime of free positrons in perfect GaN crystal (bulk) and positrons trapped at various defects.

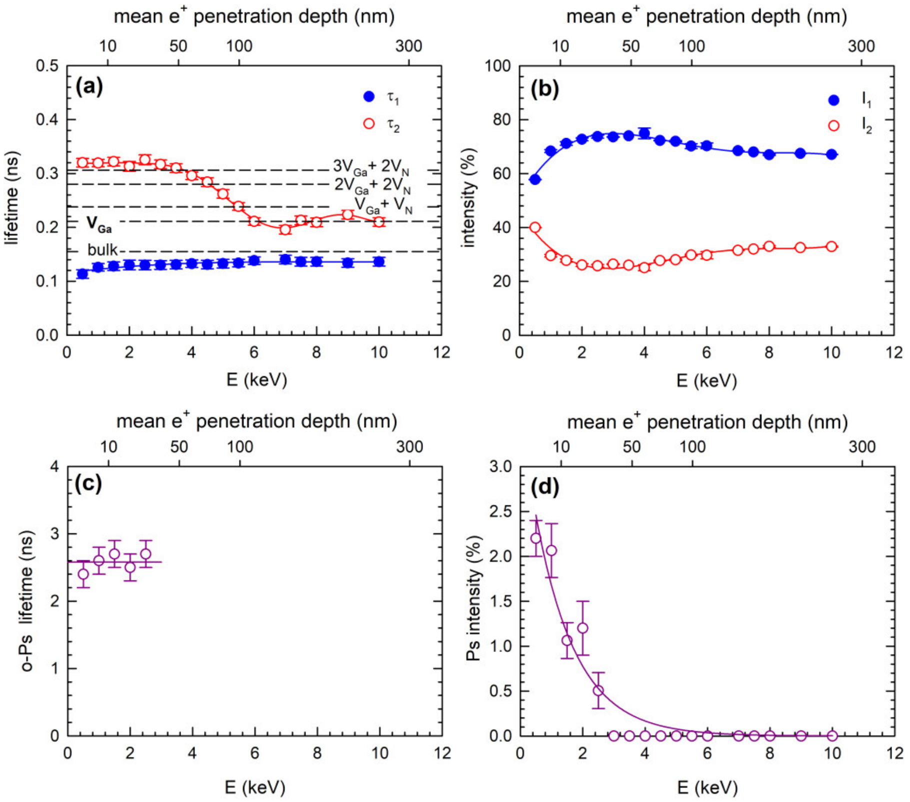

Figure S11. Results of decompositions of positron lifetime spectra for the sample TMH2 plotted as a function of the energy of incident positrons. (a) the development of lifetimes τ_1 and τ_2 of positrons annihilated in the free state and trapped at defects, respectively; (b) corresponding intensities I_1, I_2; (c) lifetime of o-Ps pick-off annihilation; (d) intensity of the o-Ps contribution. The mean positron penetration depth is shown in the upper x-axis. Dashed lines in the panel (a) indicate calculated lifetime of free positrons in perfect GaN crystal (bulk) and positrons trapped at various defects.

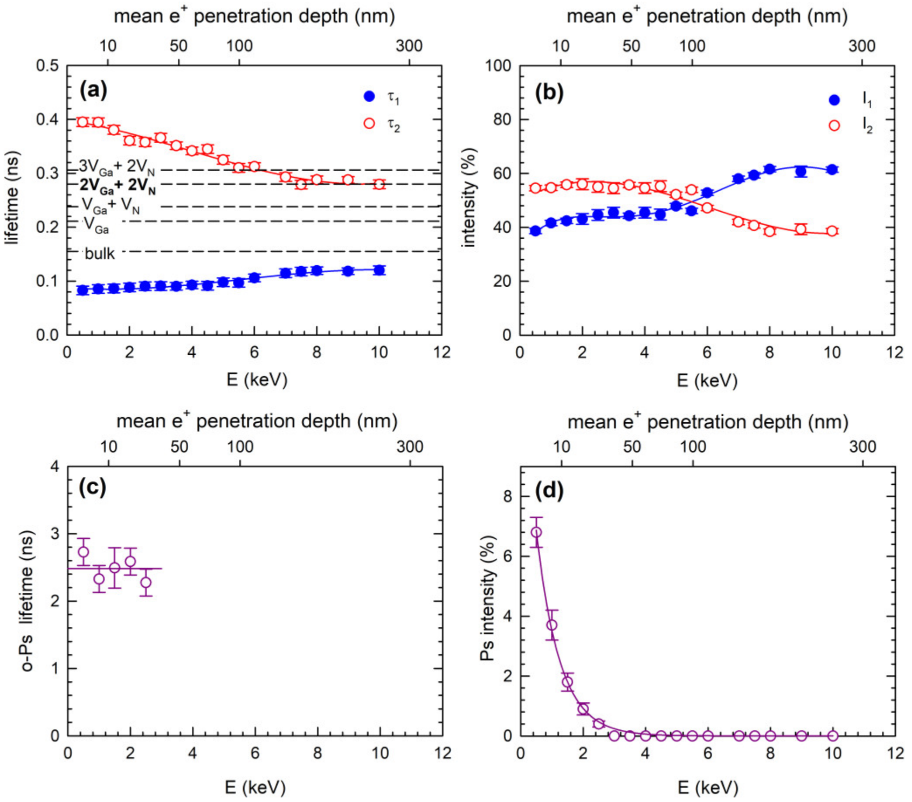

Figure S12. Results of decompositions of positron lifetime spectra for the sample TMH3 plotted as a function of the energy of incident positrons. (a) the development of lifetimes τ_1 and τ_2 of positrons annihilated in the free state and trapped at defects, respectively; (b) corresponding intensities I_1, I_2; (c) lifetime of o-Ps pick-off annihilation; (d) intensity of the o-Ps contribution. The mean positron penetration depth is shown in the upper x-axis. Dashed lines in the panel (a) indicate calculated lifetime of free positrons in perfect GaN crystal (bulk) and positrons trapped at various defects.

References

1. Čížek, J. PLRF Code for Decomposition of Positron Lifetime Spectra. *Acta Phys. Pol. A* **2020**, *137*, 177-187; 10.12693/APhysPolA.137.177.
2. Mogensen, O.E. *Positron Annihilation Chemistry*; 1st ed., Springer−Verlag: Berlin, Germany, 1995.
